# Mortality Risk Analysis of Combination Antiplatelet Therapy in Patients with Ischemic Stroke and Acute Kidney Injury: A Retrospective Cohort Analysis of the MIMIC-IV Database

**DOI:** 10.3390/diseases13050141

**Published:** 2025-05-02

**Authors:** Qiangqiang Zhou, Hongyu Xu, Shengrong Long, Wei Wei, Xiang Li

**Affiliations:** 1 Department of Neurosurgery, Zhongnan Hospital of Wuhan University, Wuhan 430071, China; qqzhou@whu.edu.cn (Q.Z.);; 2 Brain Research Center, Zhongnan Hospital of Wuhan University, Wuhan 430071, China; 3 Frontier Science Center for Immunology and Metabolism, Wuhan University, Wuhan 430072, China; 4 Medical Research Institute, Wuhan University, Wuhan 430072, China; 5 Sino-Italian Ascula Brain Science Joint Laboratory, Wuhan University, Wuhan 430071, China

**Keywords:** ischemic stroke, acute kidney injury, antiplatelet therapy, MIMIC-IV database

## Abstract

Background: Ischemic stroke (IS), a major cerebrovascular disease, is associated with high disability and mortality rates. Acute kidney injury (AKI) often complicates IS and increases in-hospital mortality. While antiplatelet agents are commonly used for IS treatment, their effectiveness in IS patients with AKI is unclear. Methods: This study, using data from the MIMIC-IV database, divided patients into non-combination (clopidogrel or ticagrelor alone) and combination (with aspirin) groups. The primary outcome was 28-day mortality, with secondary outcomes including 90-day, 1-year, and in-hospital mortality. Multivariable Cox and logistic regression models were used to analyze the relationship between antiplatelet regimens and mortality. Subgroup analyses and interaction tests were conducted. Results: Results showed the combination group had lower 28-day, 90-day, 1-year, and in-hospital mortality risks than the non-combination group (all *p* < 0.001). Subgroup analysis revealed an interaction effect by AKI stage, with combination therapy not significantly reducing mortality in severe AKI (stages 2 and 3, *p* = 0.743, *p* = 0.244). Conclusions: This study demonstrates that combination antiplatelet therapy significantly reduces 28-day, 90-day, 1-year, and in-hospital mortality risks of IS patients with AKI, suggesting its potential benefits in improving both short- and long-term clinical outcomes. However, this does not apply to patients with severe AKI, indicating heterogeneous survival benefits of combination therapy across AKI severity. Clinical decision-making should incorporate AKI stage stratification to evaluate the applicability of combination antiplatelet therapy. Further research is needed to explore the impact of AKI staging on antiplatelet therapy in IS patients.

## 1. Introduction

As the second leading cause of death globally, stroke is linked to high rates of disability and mortality1 [1,2,3]. Ischemic stroke (IS) accounts for 70% of all strokes [1], primarily manifested as cerebral infarction due to insufficient blood supply to brain tissue. Patients with IS often suffer from acute kidney injury (AKI), which is associated with a significant increase in in-hospital mortality [4]. AKI is defined as an acute deterioration of kidney function, characterized by abnormal elevation of serum creatinine and reduced urine output within a short period. AKI is frequently observed in patients with acute stroke and other critically ill patients with various diseases. Medications, fluid intake, sepsis, and contrast agents used in diagnostic processes can all lead to the occurrence of AKI [5].

Antiplatelet agents have been widely used in the therapy of individuals with IS to prevent the recurrence of ischemic events. Antiplatelet agents mainly include aspirin and P2Y12 receptor antagonists (clopidogrel and ticagrelor) [6,7]. In clinical practice, antiplatelet therapy often involves the combined or separate use of antiplatelet agents with different mechanisms of action. Multiple clinical trials based on different racial populations [7,8,9] have shown that the combined use of aspirin and P2Y12 receptor antagonists significantly reduces the risk of recurrent ischemic events. However, in specific populations of patients with IS (for example, patients carrying the CYP2C19 loss-of-function (LoF) allele [10], the clinical efficacy of the combined use of aspirin and clopidogrel is not significant. The differences in clinical efficacy of combined antiplatelet drug regimens among different populations limit the generalizability of the conclusions from previous clinical trials. Although several current studies have shown the protective effects of antiplatelet agents in AKI [11,12], there is no evidence to indicate the superiority or inferiority of combined antiplatelet drug regimens among patients suffering from IS along with AKI.

Therefore, this study analyzed patient records from the MIMIC-IV database related to intensive care to evaluate the clinical efficacy of combined antiplatelet therapy regimens in patients experiencing both IS and AKI. Our study comprehensively assessed the effects of antiplatelet therapy regimens upon short-term and long-term mortality outcomes, aiming to assist in the selection of antiplatelet therapy regimens for special patient populations in clinical practice.

## 2. Materials and Methods

### 2.1. Data Source

The dataset for this study was gathered from the Medical Information Mart for Intensive Care (MIMIC)-IV (version 2.2). This dataset encompasses de-identified clinical details of patients admitted to the intensive care unit (ICU) at Beth Israel Deaconess Medical Center (BIDMC) in Boston, Massachusetts, from 2008 to 2019. The information includes demographic characteristics, vital signs, laboratory test results, treatments, and prescription data. The author (Qiangqiang Zhou) was given permission to access the database (certificate number: 43904630). Approval for using this database was granted by the Institutional Review Boards of MIT and Beth Israel Deaconess Medical Center, with informed consent being waived.

### 2.2. Inclusion and Exclusion Criteria

#### 2.2.1. Inclusion Criteria

A total of 4195 patients with IS were initially recruited according to the International Classification of Diseases (ICD) 9/10 guidelines. Among these, 3018 individuals with a concurrent diagnosis of AKI were selected for further study. According to the KDIGO criteria, AKI was diagnosed if any of the following conditions were met: an increase in serum creatinine (Scr) of more than 26.5 μmol/L (0.3 mg/dl) within 48 h; an increase in Scr to more than 1.5 times the baseline level, confirmed or presumed to have occurred within 7 days; or urine output less than 0.5 mL/(kg·h) for more than 6 h. The AKI stage was determined based on the changes in Scr and urine output.

#### 2.2.2. Exclusion Criteria

1. Individuals admitted who are below the age of 18;
2. Individuals with an ICU duration of under 3 h;
3. Only the data from the first ICU admission were included for subjects with multiple admissions;
4. Patients who did not receive antiplatelet therapy during hospitalization;
5. Individuals diagnosed with severe liver disease, malignant tumors, or other serious illnesses.

### 2.3. Data Collection

Navicat Premium Lite 17 was deployed to retrieve patient data using Structured Query Language (SQL). The extracted patient information primarily encompassed five aspects:

1. Demographic Data: Age, sex, racial background, and body mass index (BMI).
2. Physiological Parameters within 24 h of ICU Admission: Mean arterial pressure (MAP), heart rate (HR), respiratory rate (RR), body temperature, and oxygen saturation (SpO2) measured by pulse oximetry.
3. Laboratory Parameters within 24 h of ICU Admission: Red blood cell count (RBC), platelet count (PLT), white blood cell count (WBC), blood glucose (BG), sodium and potassium levels, serum creatinine (Scr), blood urea nitrogen (BUN), and urine output.
4. Severity of Illness Scores and Comorbidities at Admission: Glasgow Coma Scale (GCS), Sequential Organ Failure Assessment (SOFA), Logistic Organ Dysfunction System (LODS), Oxford Acute Severity of Illness Score (OASIS), Acute Physiology and Chronic Health Evaluation III (APACHE III), Simplified Acute Physiology Score II (SAPS II), Systemic Inflammatory Response Syndrome (SIRS), bleeding, heart failure (HF), chronic lung disease (CLD), diabetes, hypertension, dementia, smoking, alcohol consumption, atrial fibrillation (AF), coronary atherosclerotic heart disease (CHD; carotid atherosclerosis (CAS) myocardial infarction (MI), chronic kidney disease (CKD), and history of antiplatelet drug use.

According to the Bleeding Academic Research Consortium (BARC) definition criteria, bleeding includes, but is not limited to the following: intracranial bleeding, intraocular bleeding with visual impairment, pericardial tamponade, and other life-threatening bleeding events. Additionally, clinically significant declines in hemoglobin levels (requiring laboratory confirmation) or cases necessitating substantial blood transfusion within a short timeframe (per clinical transfusion guidelines) are also encompassed within this definition.

5. Prescription Information during ICU Stay: Medication usage and therapeutic interventions, including antiplatelet drugs (aspirin, clopidogrel, ticagrelor), warfarin, new oral anticoagulants (NOAC), vasoactive drugs, mechanical ventilation (MV), thrombolysis, and continuous renal replacement therapy (CRRT).

### 2.4. Primary Outcome and Secondary Outcomes

The primary outcome of this study was the 28-day mortality rate, with 90-day mortality, 1-year mortality, and in-hospital mortality serving as secondary outcomes.

### 2.5. Statistical Analysis

For all participants, descriptive analysis was performed. Continuous variables with normal distribution were presented as mean ± standard deviation (mean ± SD), while those with skewed distribution were represented by median and interquartile range [M (Q1, Q3)]. Independent samples t-tests and Mann–Whitney U tests were used to compare differences between groups. Categorical data were expressed as counts and proportions [n (%)], and analyzed using χ^2^ tests or Fisher’s exact test. Multiple imputation was employed to handle missing data in the study. VIFs were used to assess multicollinearity among variables, with variables having a VIF exceeding 5 being excluded. To mitigate baseline imbalances, propensity score matching (PSM) was utilized to adjust for confounding factors between groups. A caliper value of 0.1 was set, and the nearest neighbor matching method was applied at a 1:1 ratio. Standardized mean differences (SMD) were used to compare differences between groups, with SMD < 0.10 indicating acceptable balance between groups. Kaplan–Meier methods were employed for survival analysis, with log-rank tests used for comparisons. Cox proportional hazards regression models were applied to assess the risk ratios (HR) and 95% confidence intervals (95% CI) for mortality outcomes (28-day, 90-day, and 1-year mortality). Logistic regression models were constructed to examine the impact of treatment regimens on in-hospital mortality. Four models were built with adjustments for confounding factors: Model 1 was the baseline model without adjustments; Model 2 adjusted for age, race, gender, BMI, and ICU admission details; Model 3 further adjusted for admission laboratory tests and disease severity scores, including HR, MBP, SpO2, RR, WBC, PLT, RBC, BG, blood potassium, blood sodium, urine volume, Scr, BUN, SOFA, LODS, OASIS, APS III, SIRS, and admission GCS; and Model 4 adjusted for comorbidities and prescription usage, including HF, CLD, diabetes, hypertension, dementia, smoking, alcohol consumption, AF, CHD, MI, CKD, history of antiplatelet drugs, statins, warfarin, NOAC, vasoactive drugs, MV, thrombolysis, and CRRT. All statistical analyses were performed using R version 4.2.2, two-tailed tests, and *p* < 0.05 was statistically significant.

### 2.6. Subgroup Analysis

Subgroup analysis was conducted according to age, gender, hypertension, diabetes, anticoagulants, atherosclerosis, AKI stage, atrial fibrillation, and bleeding. For each subgroup, Cox proportional hazards regression analysis or logistic regression was performed, and the results were visually presented using forest plots. Likelihood ratio tests were employed to detect interactions between the intervention effects and subgroup factors. A *p*-value for interaction < 0.05 indicated a significant subgroup effect, suggesting that the subgroup factor modified the effect of the intervention.

## 3. Results

### 3.1. Clinical Characteristics

**Figure 1:**
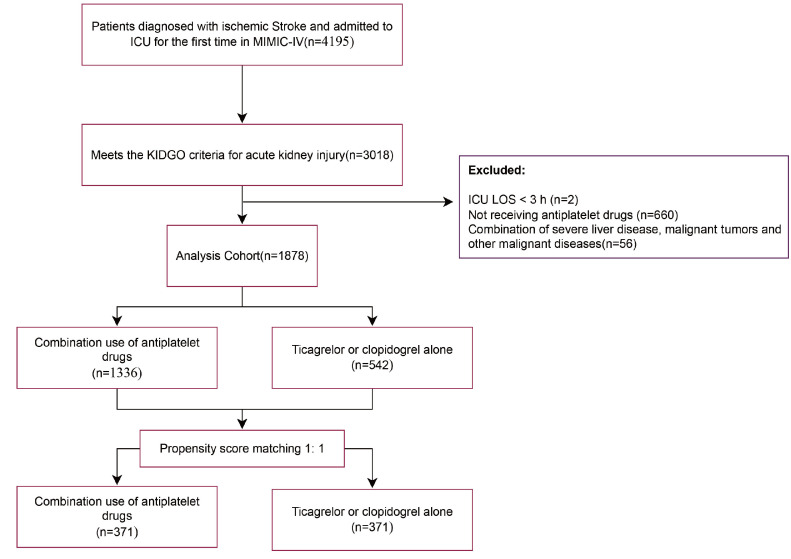
Research flowchart.

Data from 4195 IS patients were extracted from the MIMIC-IV database. After applying exclusion criteria (patients with hospitalization duration < 3 h and comorbid malignant diseases were excluded), 1878 patients with IS complicated by AKI were included in the study finally (Figure 1).

**Figure 2:**
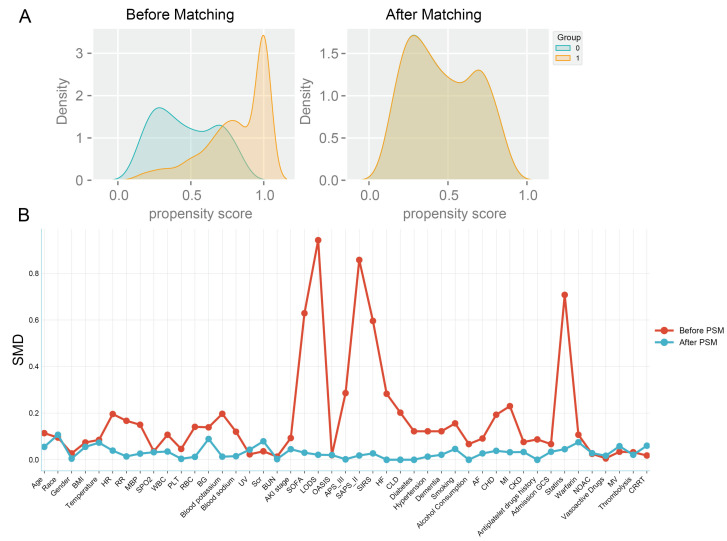
Balance of baseline characteristics between groups before and after propensity score matching, (**A**). Probability density curves before and after matching: the probability density curves of the propensity scores of the two groups intersect before matching (left panel), indicating that the propensity scores can be matched. The higher the degree of concordance of the probability density curves after matching, the better the matching effect, (**B**). Standardized Mean Difference SMD of the variables before and after the PSM, SMD < 0.1 indicates a good balance.

Table S1 presents the variance inflation factors (VIF) for the study variables, with variables having a VIF greater than 5 indicating significant collinearity and thus being excluded from the study. Table S2 displays baseline characteristics of patients. A total of 1336 patients (71.2%) were on a combination therapy of ticagrelor or clopidogrel along with aspirin during their hospital stay (combination group), while the remaining 542 patients (28.8%) did not receive aspirin (non-combination group). Table 1 demonstrates that a substantial proportion of IS patients had high-risk predisposing conditions. Notably, over 20% of patients exhibited concomitant carotid and coronary atherosclerosis. Additionally, the prevalence of atrial fibrillation reached 42.3% in the combination group and 46.8% in the non-combination group. Compared to the non-combination group, the combination group had a relatively lower mean age. Regarding routine admission examination indicators, the two groups showed different distribution patterns. The combination group had higher levels of mean arterial pressure (MBP), heart rate (HR), and respiratory rate (RR), whereas the non-combination group had higher levels of blood potassium and blood sodium. In terms of disease severity scores, the combination group had a relatively higher average OASIS. However, the combination group had a lower proportion of patients with chronic complications such as heart failure (19.7%), chronic lung disease (14.0%), myocardial infarction (10.3%), diabetes (31.9%), hypertension (75.1%), CHD (12.0%), and other conditions. To reduce confounding bias, propensity score matching was applied to the covariates, resulting in 371 matched pairs after 1:1 matching. The probability density curves of the propensity scores before and after the matching process are shown in Figure 2A. The baseline characteristics of the patients in both groups were similar, with the standardized mean differences (SMD) of the variables before and after matching reflecting the balance of the baseline data between groups (Figure 2B).

### 3.2. Primary and Secondary Outcomes

**Figure 3:**
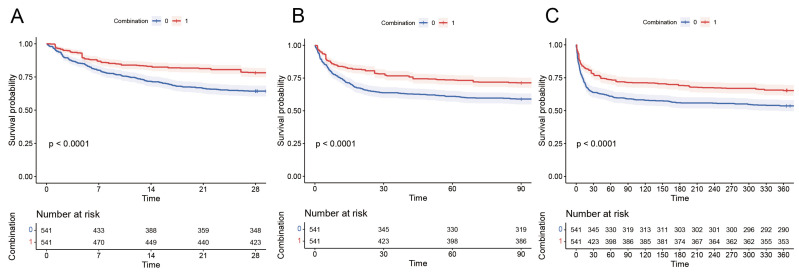
Kaplan–Meier survival curves of combination group and non-combination group: (**A**) 28-day mortality, (**B**) 90-day mortality, and (**C**) 1-year mortality.

The Kaplan–Meier curves demonstrated that compared to the patients in the non-combination group, the combination group had significantly lower 28-day, 90-day, and 1-year mortality rates. (log-rank test, *p* < 0.0001, Figure 3).

The development of four multivariable Cox regression models was undertaken to assess the independent impact of two treatment regimens on mortality outcomes, with respective hazard ratios (HR) and 95% confidence intervals (CI) presented in Table 1 and Table S3. The unadjusted Model 1 Cox regression results indicated that individuals in the combination group had a significantly reduced 28-day mortality rate (HR 0.56, 95% CI 0.44–0.70, *p* < 0.001). For secondary outcomes, the combination group also showed significantly lower risks for 90-day and 1-year mortality, as well as in-hospital mortality (HR 0.56, 95% CI 0.44–0.70, *p* < 0.001; HR 0.63, 95% CI 0.51–0.77, *p* < 0.001; HR 0.66, 95% CI 0.55–0.80, *p* < 0.001; HR 0.51, 95% CI 0.39–0.60, *p* < 0.001). In the extended multivariable Cox regression models, the combination group consistently exhibited a lower risk of death, emphasizing the potential benefits of combined antiplatelet therapy in reducing the mortality risk among patients with IS complicated by AKI.

Given that atrial fibrillation (AF) is an independent risk factor for stroke prognosis, adjustment for its confounding effects on the therapeutic outcomes of antiplatelet therapy was required. A separate survival analysis conducted in AF patients (n = 1605) demonstrated through Kaplan–Meier curves that combination antiplatelet therapy significantly improved survival outcomes even in this population (Figure S1, log-rank test, *p* < 0.001).

### 3.3. Subgroup Analysis and Interaction Analysis

**Figure 4:**
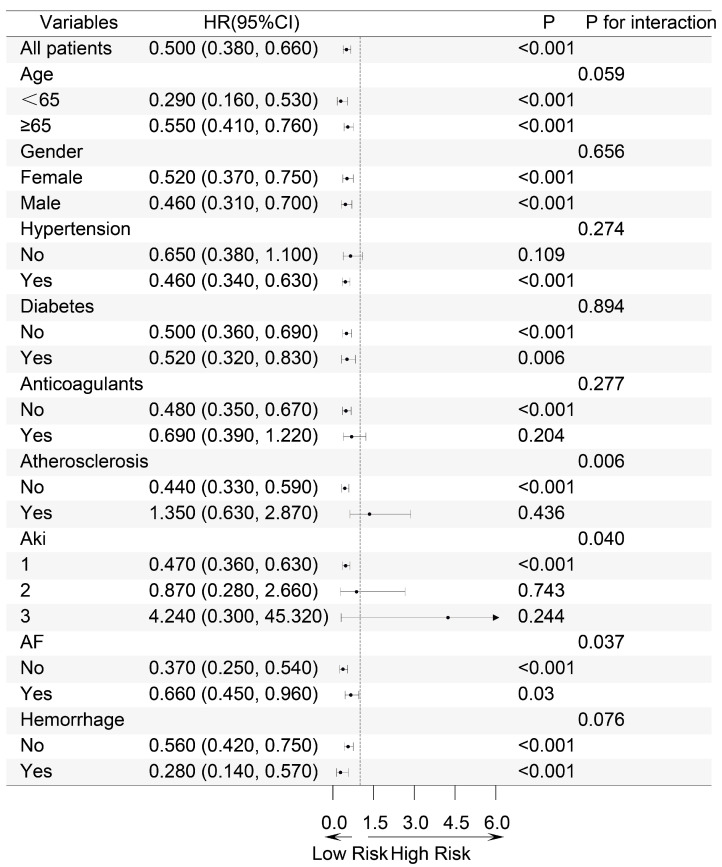
Subgroup Forest Map of 28-day all-cause mortality. Based on age, gender, hypertension, diabetes, anticoagulants, atherosclerosis, AKI stage, atrial fibrillation and bleeding. HR: Hazard Ratio, CI: Confidence Interval.

Patients were stratified into different subgroups based on age, gender, hypertension, diabetes, anticoagulants, atherosclerosis, AKI stage, atrial fibrillation, and bleeding to explore the impact of exposure on mortality outcomes across these subgroups. The results of the subgroup analysis for the 28-day mortality outcome are presented in a forest plot (Figure 4).

Significant interactions were observed in the subgroups of atherosclerosis, atrial fibrillation, and AKI stage. Notably, in the AKI stage subgroup, a significant difference in the 28-day mortality risk was observed across different stages of AKI (*p* for interaction 0.04). For patients diagnosed with AKI stage 1, the implementation of combination therapy resulted in a 47% reduction in mortality risk. (HR 0.53, 95% CI 0.36–0.63, *p* < 0.001), whereas this benefit was not evident in patients with stages of AKI 2 and 3. Consistent results were also observed in the subgroup analyses for secondary mortality outcomes (Figures S2–S4). This suggests that for IS patients with more severe stages of AKI, the combined use of antiplatelet drugs does not improve the mortality risk compared to the use of clopidogrel or ticagrelor alone.

### 3.4. Outcomes in Mild and Severe AKI Subgroups

**Figure 5:**
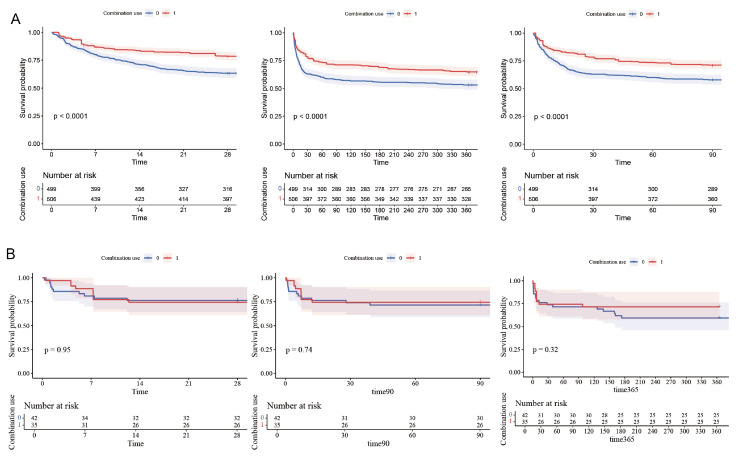
Kaplan–Meier survival curves for 28-day, 90-day and 1-year mortality in MAKI and SAKI groups. (**A**) MAKI group. (**B**) SAKI group.

Subsequently, the patients were divided into two groups according to the stage of AKI they were experiencing. Patients with AKI stage 1 were defined as having mild AKI (MAKI), while those with stages 2 and 3 were categorized as having severe AKI (SAKI). Kaplan–Meier (KM) curves were used to display the 28-day, 90-day, and 1-year mortality rates for patients with mild and severe AKI, respectively (Figure 5).

The results of the multivariable Cox proportional hazards analysis, as presented in Table 2, indicated that patients in the mild AKI (MAKI) group had a significantly reduced 28-day mortality rate (HR 0.59, 95% CI 0.46–0.76, *p* < 0.001). Consistent findings were observed in the Cox regression models for secondary outcomes in the MAKI group. In contrast, for patients with severe AKI (SAKI), no significant differences in the impact of the two treatment regimens on primary and secondary mortality outcomes were observed. These findings align with the results depicted in the Kaplan–Meier (KM) curves. This suggests that for IS patients with more severe stages of AKI, the combined use of antiplatelet drugs does not confer a survival benefit over the use of clopidogrel or ticagrelor alone.

For patients with AKI complicated by IS, we conducted mediation analysis (Table S4) considering potential bleeding events influenced by antiplatelet therapy that might mediate clinical outcomes. Bootstrap testing revealed no significant indirect mediation effects of bleeding events across all primary and secondary outcomes (*p* = 0.570, *p* = 0.496, *p* = 0.305, *p* = 0.840).

Furthermore, in addition to the AKI-complicated IS cohort, we analyzed outcomes in IS patients without AKI. Propensity score matching-adjusted survival analysis (Figure S5) demonstrated that combination antiplatelet therapy significantly prolonged 28-day, 90-day, and 1-year survival durations (all *p* < 0.001).

## 4. Discussion

In the treatment of IS, combination antiplatelet therapy (P2Y12 receptor antagonists combined with aspirin) has been widely used. Previous trials such as CHANCE [8], POINT [7], and THALES [9] have demonstrated that combination antiplatelet therapy reduces the risk of new ischemic vascular events compared to monotherapy with antiplatelet drugs and can prevent myocardial infarction and ischemic events. However, the efficacy of combination antiplatelet therapy varies in certain populations; for instance, clopidogrel is less effective in patients carrying the CYP2C19 LOF alleles [10].

In this study, the clinical efficacy of antiplatelet combination therapy versus monotherapy in patients with IS who also experienced AKI was investigated. After propensity score matching and adjustment for confounding factors using multiple models, we determined that combination antiplatelet therapy significantly reduced the 28-day, 90-day, 1-year, and in-hospital mortality risks.

Baseline characteristics revealed a high prevalence of atherosclerotic disease and atrial fibrillation (>40%) as comorbid conditions among ischemic stroke patients with concurrent acute kidney injury. Both atherosclerosis severity and atrial fibrillation are established risk factors for cerebral infarction. Clinical evidence confirms that stroke patients with comorbid atrial fibrillation typically exhibit poorer clinical outcomes. Therefore, a separate analysis was conducted to evaluate the prognostic impact of antiplatelet regimens specifically in this high-risk subgroup. The results demonstrated that combination antiplatelet therapy significantly reduced mortality risk even when restricted to the atrial fibrillation cohort (*p* < 0.001).

However, subsequent subgroup analyses revealed that the severity of AKI may influence the clinical benefits of this therapeutic regimen. The Cox proportional hazards model revealed that in patients with mild renal impairment, combination antiplatelet therapy was associated with a significantly lower risk of mortality. Similarly, in ischemic stroke patients without renal impairment, this therapy was linked to better prognosis. But in severe renal impairment cases, no statistically significant difference was found between the two treatment regimens. The survival analyses for patients with mild and severe AKI, along with multivariable Cox proportional hazards analyses and Kaplan–Meier survival curves, corroborated the subgroup analysis results. That is, in stroke patients, combination antiplatelet therapy improved the prognosis of those with mild AKI compared to single antiplatelet drug use but offered no benefits for severe AKI patients.

Antiplatelet therapy has been shown to improve AKI in animal models and some clinical studies, indicating that platelets are an important target in the pathophysiology of AKI. Two mouse studies reported the protective effects of clopidogrel on AKI. Clopidogrel increases the total antioxidant capacity of the kidneys to eliminate renal tubular cell apoptosis and reduces renal NET formation to prevent kidney ischemia–reperfusion injury (I/R)-induced AKI [13,14]. Regarding the clinical effects of aspirin in AKI patients, there are conflicting results. Several studies have shown that the use of aspirin significantly reduces the risk of postoperative AKI. However, the POISE-2 trial results indicated that preoperative ASA administration did not reduce the risk of AKI in patients undergoing major non-cardiac surgery but was instead associated with an increased incidence of major bleeding [15]. This may be due to bleeding as an independent risk factor for AKI affecting the protective effect of aspirin on AKI [16,17].

In AKI patients, activated platelets contribute to the pathologic process of AKI by affecting renal hemodynamics and renal inflammation. Activated platelets release mediators that increase endothelial permeability, such as platelet-activating factor (PAF) [18] and 5-hydroxytryptamine (5-HT) [19]. Platelets promote thrombus formation and the release of coagulation mediators, further leading to the deterioration of renal microcirculation [20]. Moreover, platelets cause renal tubular epithelial cell injury by stimulating endothelial cells and recruiting and activating white blood cells during inflammatory responses [21], ultimately leading to renal functional impairment.

Based on the protective effects of antiplatelet therapy in IS and AKI patients, the benefits of combination antiplatelet therapy in patients with both conditions could be anticipated. However, in our study, the benefits of combination antiplatelet therapy did not surpass those of P2Y12 antagonist monotherapy. This may be attributed to the heterogeneity of platelet reactivity and the increased bleeding risk associated with combination antiplatelet drugs.

The severity of renal impairment was found to significantly influence clinical outcomes following antiplatelet therapy, potentially attributable to differential therapeutic responsiveness in severe renal impairment patients, which may contribute to elevated mortality risk.

We postulate that hemorrhagic events may act as a mediator between antiplatelet therapy and mortality in ischemic stroke patients with severe renal impairment, whereas in those with mild renal impairment, mortality reduction appears primarily driven by antithrombotic efficacy, with hemorrhagic complications demonstrating minimal mediating effects. Mediation analysis employing the Bootstrap method revealed no significant mediating role of hemorrhagic events in mortality risk across both primary and secondary endpoints in severe renal impairment stroke cohorts (*p =* 0.570, *p =* 0.496, *p =* 0.305, *p =* 0.840). These results suggest that the prognostic impact of renal impairment severity on antiplatelet therapy efficacy may be mediated through mechanisms independent of hemorrhagic complications.

In clinical practice, it is acknowledged that certain populations exhibit variability in the effectiveness of antiplatelet therapy, known as variable platelet reactivity (VPR) [22]. High on-treatment platelet reactivity (HPR) after antiplatelet therapy indicates a higher risk of thrombotic events [23], while low on-treatment platelet reactivity (LPR) corresponds to a higher risk of bleeding events [24].

Factors influencing the mechanism of action of antiplatelet drugs can lead to the heterogeneity of platelet reactivity.

Firstly, the severity of renal impairment correlates with abnormal metabolism of antiplatelet drugs, particularly in patients with advanced renal disease. In addition, in patients with severe renal impairment, alterations in platelet pharmacological targets occur, leading to diverse platelet reactions. Changes in renal function can affect drug pharmacokinetics, including both renally cleared and non-renally cleared drugs [25]. Numerous studies indicate that chronic kidney disease reduces renal drug excretion. Moreover, accumulated uremic toxins may affect the activity and/or expression of drug-metabolizing enzymes and transporters. Research in rodent models of renal failure shows that the mRNA and protein expression of many members of the cytochrome P450 (CYP) gene family, as well as the ATP-binding cassette (ABC) and solute carrier (SLC) gene families of drug transporters, is reduced. In uremic patients, the impaired excretion of renal toxins leads to the accumulation of substances that interfere with transcriptional activation. This results in down-regulation of gene expression mediated by pro-inflammatory cytokines and directly inhibits the activity of cytochrome P450 and drug transporters [26].

Aspirin irreversibly inhibits platelet cyclooxygenase (COX-1), reducing the production of thromboxane A2 (TXA2) and thereby inhibiting platelet aggregation. Thienopyridine drugs such as clopidogrel and ticagrelor [27] act on the P2Y12 receptor to inhibit platelet aggregation, with irreversible and reversible binding to the P2Y12 receptor, respectively, blocking the activation of GPⅡb/Ⅲa by ADP. External and internal factors acting on these pharmacological targets lead to the VPR phenomenon. External factors include patient race [28], sex, age [29], drug interactions [30], disease severity [31], and comorbidities [32]. Internal factors include genes related to drug absorption and metabolism [33] and polymorphisms of platelet receptor genes [34]. Taking clopidogrel as an example, genetic loci involved in pharmacokinetics and pharmacodynamics all affect platelet reactivity, including the ABCB1 gene related to drug absorption, the CYP2C19 gene for drug metabolism, and the platelet action targets P2Y12 and ITGB3 genes. As the genetic basis for aspirin’s variable platelet reactivity, genetic variations in COX-1-dependent and -independent platelet activation pathways have been proposed to explain the differences in aspirin reactivity among individuals [35]. Many studies have reported specific gene variations in COX-1, with Halushka reporting that two SNPs (A-842G and C50T) in a cohort of healthy individuals were in linkage disequilibrium, and individuals with the G/T haplotype showed greater inhibition of prostaglandin H formation and arachidonic acid-induced aggregation [36].

It is noteworthy that the presence and severity of renal impairment may also affect platelet reactivity, which may partly explain the different effects of combination antiplatelet therapy in patients with different degrees of renal injury in this study. In the case of renal impairment, the efficacy of clopidogrel and aspirin is affected, increasing mortality and the incidence of cardiovascular events [37].

Moreover, in cases of renal impairment, platelet activity and responsiveness to drugs are compromised, potentially increasing cardiovascular event and mortality rates [38]. Uremic patients show altered platelet activity and structure. During dialysis, blood–film contact in uremia causes PF4 and β-thromboglobulin release from platelets due to granule–membrane defects, activating platelets [39].

Patients with chronic kidney disease (CKD) have a higher prevalence of HPR [40], impaired P2Y12 inhibition [41], and reduced bioavailability of clopidogrel’s active metabolite [42]. Similarly, impaired aspirin reactivity has been observed in CKD patients [43]. A. Polzin et al. further demonstrated that the degree of renal impairment in CKD is associated with impaired antiplatelet effects of aspirin [44]. Possible reasons include the concentration of von Willebrand factor in platelets of CKD patients, the expression of activated glycoprotein IIb/IIIa, and the increase in platelet-derived microparticles46 [45].

Given the significant heterogeneity in P2Y12 receptor reactivity among individuals, optimization strategies for antiplatelet therapy combining P2Y12 receptor inhibitors (e.g., clopidogrel) with aspirin have garnered considerable attention. Although combination antiplatelet therapy with aspirin and clopidogrel demonstrates consistent efficacy across diverse clinical settings, patients receiving this regimen continue to exhibit a high residual risk of ischemic events. To reduce the incidence of high platelet reactivity (HPR) during treatment, multiple approaches have been proposed, including the use of more potent P2Y12 receptor inhibitors. Studies have shown that compared to clopidogrel, ticagrelor administered as a loading dose followed by a standard maintenance dose is associated with a significant reduction in long-term ischemic events in patients with acute coronary syndrome [46]. Furthermore, low-dose ticagrelor regimens appear to offer superior safety and efficacy profiles while maintaining equivalent ischemic protection [47]. Notably, in patients with end-stage renal disease undergoing hemodialysis, low-dose ticagrelor exerts a more pronounced platelet inhibitory effect compared to clopidogrel [48]. Additionally, precision antiplatelet therapy guided by CYP2C19 genotyping has enhanced antiplatelet treatment efficacy in clinical practice [49]. Patients carrying CYP2C19 LoF alleles exhibit reduced clopidogrel metabolism, which is associated with diminished platelet inhibition and an elevated risk of thrombotic events [50]. Clinical trials have demonstrated that ticagrelor provides greater anti-ischemic benefits in patients harboring CYP2C19 LoF alleles [51].

The CHANCE-2 trial results demonstrated that among Chinese patients with minor ischemic stroke or transient ischemic attack (TIA) carrying CYP2C19 LoF alleles, ticagrelor was associated with a modestly reduced risk of stroke at 90 days compared to clopidogrel. And ticagrelor was linked to a higher incidence of total bleeding events [52].

As a retrospective study, our results have some limitations. First, the possibility of selection bias cannot be overlooked. The patient population in this study comes from the MIMIC database, which records patients from intensive care units. The study group may not fully represent a broader patient population, which may limit the generalizability of the study results. Future studies should expand the scope to include patient populations from different sources. Second, although multiple measures were used to reduce the impact of confounding factors, the presence of unmeasured confounding variables may still affect the study conclusions. Third, as a retrospective study, it is not possible to determine the exact causal relationship between the differences in treatment regimens and mortality. Finally, due to the particularity of database data recording, there are some missing data, including new adverse events during hospitalization, such as hypersensitivity reactions, which limits the comprehensiveness of the clinical efficacy of different antiplatelet drug regimens in our study.

## 5. Conclusions

In summary, this study demonstrates that combination antiplatelet therapy significantly reduces 28-day, 90-day, 1-year, and in-hospital mortality risks of IS patients with AKI, suggesting its potential benefits in improving both short- and long-term clinical outcomes. However, this does not apply to patients with severe AKI, indicating heterogeneous survival benefits of combination therapy across AKI severity. Clinical decision-making should incorporate AKI stage stratification to evaluate the applicability of combination antiplatelet therapy. Further research is needed to explore the impact of AKI staging on antiplatelet therapy in IS patients.

## Figures and Tables

**Table 1:** Multi-model regression analysis of primary outcomes.

	28-Day Mortality		
	HR	95% CI	*p*
**Model 1**			
Non-combination	1.00	Reference	
Combination	0.56	0.44–0.70	<0.001
**Model 2**			
Non-combination	1.00	Reference	
Combination	0.55	0.44–0.71	<0.001
**Model 3**			
Non-combination	1.00	Reference	
Combination	0.58	0.45–0.75	<0.001
**Model 4**			
Non-combination	1.00	Reference	
Combination	0.58	0.45–0.75	<0.001

Model 1: crude. Model 2: adjust: age, race, gender, BMI, ICU IOS. Model 3: adjust for variables in Model 2 plus temperature, admission GCS, APS III, BG, blood potassium, blood sodium, BUN, HR, LODS, MBP, OASIS, PLT, RBC, RR, Scr, SIRS, Spo2, SOFA, WBC, UV. Model 4: adjust for variables in Model 3 plus AF, alcohol consumption, antiplatelet drugs history, CHD, CLD, CKD, CRRT, dementia, diabetes, HF, hypertension, MI, MV, smoking, statins, thrombolysis, warfarin, NOAC, vasoactive drugs. HR: hazard ratio, OR: odds ratio, CI: confidence interval.

**Table 2:** Regression Analysis of Outcomes in Mild and Severe AKI Subgroups.

	28-Day Mortality			90-Day Mortality			1-Year Mortality			In-Hospital Mortality		
	HR	95% CI	*p*	HR	95% CI	*p*	HR	95% CI	*p*	OR	95% CI	*p*
**MAKI**												
Non-combination	1.00	Reference		1.00	Reference		1.00	Reference		0.51	0.39–0.68	
Combination	0.59	0.46–0.76	<0.001	0.66	0.52–0.83	<0.001	0.69	0.56–0.86	<0.001	0.47	0.32–0.67	<0.001
**SAKI**												
Non-combination	1.00	Reference		1.00	Reference		1.00	Reference		1.00	Reference	
Combination	1.87	0.49–7.09	0.139	1.05	0.31–3.59	0.531	0.86	0.31–2.31	0.917	0.72	0.04–11.50	0.611

MAKI, mild acute kidney injury; SAKI, severe acute kidney injury.

## Data Availability

All data analyzed in this study can be obtained through the MIMIC database.

## Supplementary Materials

The following supporting information can be downloaded at: https://www.mdpi.com/article/10.3390/diseases13050141/s1, Figure S1: Kaplan Meier survival curves of combination group and non-combination group in patients with atrial fibrillation; Figure S2: Subgroup forest map of 90-day mortality; Figure S3: Subgroup forest map of 1-year mortality; Figure S4: Subgroup forest map of in-hospital mortality.; Figure S5: Kaplan Meier survival curves of combination group and non-combination group in patients without acute kidney injury. Table S1: The respective factors of variance inflation for the variables studied; Table S2: Patient demographics and baseline characteristics; Table S3: Multi-model regression analysis of secondary outcomes; Table S4: Mediating effect analysis of Bleeding in MAKI combined with IS patients.

## Abbreviations

The following abbreviations are used in this manuscript:

IS	Ischemic stroke
AKI	Acute kidney injury
SMD	Standardized Mean Difference
LOS	Length of Stay
VIF	variance inflation factor
BG	Blood glucose
UV	Urine volume
GCS	Glasgow Coma Scale
SOFA	Sequential Organ Failure Assessment
LODS	Logistic Organ Dysfunction System
OASIS	Oxford Acute Severity of Illness Score
APS III	Acute Physiology and Chronic Health Evaluation III
SAPS II	Simplified Acute Physiology Score II
SIRS	Systemic Inflammatory Response Syndrome
CLD	Chronic Lung Disease
MI	Myocardial Infarction
MV	Mechanical Ventilation
CRRT	Continuous Renal Replacement Therapy
