# Supplementary material for: Mortality Risk Analysis of Combination Antiplatelet Therapy in Patients with Ischemic Stroke and Acute Kidney Injury: A Retrospective Cohort Analysis of the MIMIC-IV Database
Source: Diseases. 2025 May 2;13(5):141. doi: 10.3390/diseases13050141 (PMC12110695; doi:10.3390/diseases13050141)
Supplement: Supplementary file 1 [file diseases-13-00141-s001.zip › diseases-3538666-supplementary.pdf]

Supplementary Material

Table S1. The respective factors of variance inflation for the variables studied.

**Variables**	**VIF**
Age	3.002
Race	1.902
Gender	1.220
BMI	1.190
Temperature	1.326
HR	1.584
RR	1.440
MBP	1.317
SPO2	1.296
WBC	1.416
PLT	1.345
RBC	1.319
BG	1.309
Blood potassium	2.343
Blood sodium	2.367
UV	1.268
Scr	1.840
BUN	1.996
Admission GCS	1.353
AKI	1.203
SOFA	3.094
LODS	4.562
OASIS	2.471
APS_III	4.946
SAPS_II	6.350
SIRS	1.335
HF	1.330
CLD	1.180
Diabetes	1.379
Hypertension	1.337
Dementia	1.180
Smoking	1.113
Alcohol Consumption	1.113
AF	1.528

CHD	1.357
CAA	1.337
Bleeding	1.335
MI	1.245
CKD	1.522
Antiplatelet drugs history	1.225
Statins	1.324
Warfarin	1.411
NOAC	1.117
Vasoactive Drugs	1.117
MV	1.685
Thrombolysis	1.146
CRRT	1.390

VIF, variance inflation factor; AF, Atrial Fibrillation; APS III, Acute Physiology and Chronic Health Evaluation III; BG, Blood Glucose; BUN, Blood Urea Nitrogen; CAA, Carotid Arterial Atherosclerosis; CHD, Coronary atherosclerotic heart disease; CKD, Chronic kidney disease; CLD, Chronic Lung Disease; CRRT, Continuous Renal Replacement Therapy; GCS, Glasgow Coma Scale; HF, heart failure; LODS, Logistic Organ Dysfunction System; LOS, Length of Stay; MI, Myocardial Infarction; MV, Mechanical Ventilation; NOAC, New-oral-anticoagulants; OASIS, Oxford Acute Severity of Illness Score; PLT, platelet count; RBC, red blood cell count; SAPS II, Simplified Acute Physiology Score II; Scr, Serum creatinine; SIRS, Systemic Inflammatory Response Syndrome; SMD, Standardized Mean Difference; SOFA, Sequential Organ Failure Assessment; UV, Urine volume; WBC, white blood cell count.

Table S2 Patient demographics and baseline characteristics.

	Before PSM				After PSM			
**Variables**	**Non-combination** (n=542)	**Combination** (n=1336)	***p***	**SMD**	**Non-combination**(n=371)	**Combination**(n=371)	***p***	**SMD**
Age	69.4±17.0	71.2±14.4	0.030	0.114	70.7±16.0	69.8±16.3	0.457	0.055
Race, n (%)			0.452	0.095			0.717	0.107
White	316 (58.3)	814 (60.9)			218 (58.8)	215 (58.0)		
Black	59 (10.9)	155 (11.6)			45 (12.1)	51 (13.7)		
Asian	20 (3.7)	32 (2.4)			16 (4.3)	10 (2.7)		
Hispanic	19 (3.5)	40 (3.0)			13 (3.5)	11 (3.0)		
Other	128 (23.6)	295 (22.1)			79 (21.3)	84 (22.6)		
Gender, n (%)			0.598	0.027			0.941	0.005
Female	273 (50.4)	655 (49.0)			191 (51.5)	192 (51.8)		
Male	269 (49.6)	681 (51.0)			180 (48.5)	179 (48.2)		
BMI	26.7 (25.4, 26.8)	26.7 (25.4, 28.7)	0.022	0.074	26.0±7.2	26.5±8.8	0.458	0.055
Temperature, °C	37.0±0.7	36.9±0.5	0.120	0.085	36.9±0.7	37.0±0.5	0.323	0.073

Variables	Before PSM				After PSM			
	**Non-combination** (n=542)	**Combination** (n=1336)	***p***	**SMD**	**Non-combination**(n=371)	**Combination**(n=371)	***p***	**SMD**
HR, time/min	84.5±16.1	81.5±14.6	<0.001	0.196	83.2±15.7	83.8±15.4	0.591	0.039
RR, time/min	19.8±4.2	19.1±3.7	0.002	0.167	19.7±4.2	19.7±3.7	0.847	0.014
MBP, mmHg	92.8±19.5	89.9±19.7	0.003	0.150	92.3±19.8	91.8±20.6	0.728	0.026
SPO2, %	97.2±2.2	97.2±2.1	0.481	0.036	97.2±2.0	97.2±2.0	0.663	0.032
WBC,10^9/L	11.2 (8.4, 14.3)	10.9 (8.1, 14.1)	0.185	0.107	10.6 (8.0, 13.7)	11.3 (8.5, 14.2)	0.111	0.035
PLT, 10^9/L	208.5 (158.0, 261.5)	202.0 (154.0, 259.0)	0.284	0.046	207.0 (157.5, 254.5)	203.0 (155.0, 252.0)	0.748	0.004
RBC, 10^9/L	3.9±0.8	3.8±0.8	0.006	0.141	3.9±0.7	3.8±0.8	0.874	0.012
BG, mg/dL	135.5 (110.0, 173.0)	126.5 (106.0, 160.0)	0.002	0.139	133.0 (108.0, 177.0)	130.0 (105.0, 161.5)	0.204	0.089
Blood potassium, mmol/L	3.9±1.0	4.1±0.9	<0.001	0.197	4.0±0.9	4.0±0.9	0.857	0.013
Blood sodium, mmol/L	134.9±27.6	137.6±16.3	0.032	0.120	136.8±22.2	137.1±19.7	0.835	0.015
UV, ml/day	1302.5 (795.0, 2118.8)	1390.0 (885.0, 2075.0)	0.178	0.023	1250.0 (771.0, 2076.0)	1433.0 (887.5, 2087.5)	0.046	0.043
Scr, mg/dL	1.0 (0.8, 1.4)	1.0 (0.8, 1.4)	0.770	0.036	1.0 (0.8, 1.4)	1.0 (0.8, 1.4)	0.652	0.079
BUN, mg/dL	7.0 (0.0, 7.0)	7.0 (0.0, 7.0)	0.999	0.013	7.0 (0.0, 7.0)	7.0 (0.0, 7.0)	0.916	0.003
Admission GCS	13.9±2.3	13.8±2.6	0.175	0.067	13.9±2.2	13.8±2.6	0.640	0.034
AKI, n (%)			0.502	0.093			0.591	0.045
1	500 (92.3)	1245 (93.2)			338 (91.1)	342 (92.2)		
2	38 (7.0)	72 (5.4)			30 (8.1)	27 (7.3)		
3	4 (0.7)	19 (1.4)			3 (0.8)	2 (0.5)		
SOFA	4.0 (2.0, 6.0)	4.0 (2.0, 6.0)	0.629	0.000	3.0 (2.0, 6.0)	3.0 (2.0, 6.0)	0.592	0.030
LODS	4.0 (2.0, 6.0)	4.0 (2.0, 6.0)	0.943	0.023	4.0 (2.0, 6.0)	4.0 (2.0, 6.0)	0.989	0.021
OASIS	34.5±8.4	33.5±8.3	0.019	0.119	33.7±8.4	33.6±8.1	0.783	0.020
APS_III	41.0 (31.0, 55.0)	40.0 (30.0, 54.0)	0.286	0.088	40.0 (30.0, 54.0)	41.0 (31.0, 55.0)	0.682	0.002
SAPS_II	35.0 (28.0, 44.0)	36.0 (29.0, 44.0)	0.858	0.019	35.0 (27.0, 45.0)	36.0 (29.0, 43.0)	0.838	0.018

Variables	Before PSM				After PSM			
	**Non-combination** (n=542)	**Combination** (n=1336)	***p***	**SMD**	**Non-combination**(n=371)	**Combination**(n=371)	***p***	**SMD**
SIRS	2.0 (2.0, 3.0)	2.0 (2.0, 3.0)	0.596	0.032	2.0 (2.0, 3.0)	2.0 (2.0, 3.0)	0.770	0.027
HF, n (%)	107 (19.7)	428 (32.0)	<0.001	0.283	88 (23.7)	88 (23.7)	1.000	0.000
CLD, n (%)	76 (14.0)	290 (21.7)	<0.001	0.202	65 (17.5)	65 (17.5)	1.000	0.000
Diabetes, n (%)	173 (31.9)	504 (37.7)	0.018	0.122			1.000	0.000
Hypertension, n (%)	407 (75.1)	1071 (80.2)	0.015	0.122	296 (79.8)	294 (79.2)	0.856	0.013
Dementia, n (%)	43 (7.9)	66 (4.9)	0.012	0.122	26 (7.0)	28 (7.5)	0.777	0.021
Smoking, n (%)	30 (5.5)	129 (9.7)	0.004	0.156	24 (6.5)	20 (5.4)	0.534	0.046
Alcohol Consumption, n (%)	22 (4.1)	38 (2.8)	0.175	0.067	11 (3.0)	11 (3.0)	1.000	0.000
AF, n (%)	229 (42.3)	625 (46.8)	0.074	0.091	165 (44.5)	160 (43.1)	0.711	0.027
CHD-, n (%)	65 (12.0)	253 (18.9)	<0.001	0.193	56 (15.1)	51 (13.7)	0.601	0.038
CAA, n (%)	70 (13.0)	347 (26.3)	<0.001	0.034	50 (13.6)	53 (14.5)	0.597	0.024
Bleeding, n (%)	81 (15.0)	157 (11.8)	0.005	0.096	53 (14.2)	48 (13.0)	0.460	0.034
MI, n (%)	56 (10.3)	245 (18.3)	<0.001	0.230	49 (13.2)	45 (12.1)	0.659	0.032
CKD, n (%)	103 (19.0)	295 (22.1)	0.139	0.076	79 (21.3)	74 (19.9)	0.650	0.033
Antiplatelet drugs history, n (%)	38 (7.0)	66 (4.9)	0.075	0.087	29 (7.8)	29 (7.8)	1.000	0.000
Statins, n (%)	256 (47.2)	1061 (79.4)	<0.001	0.708	245 (66.0)	237 (63.9)	0.538	0.045
Warfarin, n (%)	165 (30.4)	474 (35.5)	0.037	0.107	124 (33.4)	111 (29.9)	0.305	0.075
NOAC, n (%)	78 (14.4)	204 (15.3)	0.629	0.025	64 (17.3)	68 (18.3)	0.701	0.028
Vasoactive Drugs, n (%)	11 (2.0)	26 (1.9)	0.906	0.006	10 (2.7)	9 (2.4)	0.816	0.017
MV, n (%)	173 (31.9)	448 (33.5)	0.500	0.034	123 (33.2)	113 (30.5)	0.431	0.058
Thrombolysis, n (%)	38 (7.0)	105 (7.9)	0.530	0.032	27 (7.3)	25 (6.7)	0.774	0.021
CRRT, n (%)	7 (1.3)	20 (1.5)	0.735	0.018	4 (1.1)	2 (0.5)	0.682	0.060

AF, Atrial Fibrillation; APS III, Acute Physiology and Chronic Health Evaluation III;BG, Blood Glucose; BUN, Blood Urea Nitrogen; CAA, Carotid Arterial Atherosclerosis; CHD, Coronary atherosclerotic heart disease; CKD, Chronic kidney disease; CLD, Chronic Lung Disease; CRRT, Continuous Renal Replacement Therapy; GCS, Glasgow Coma Scale; HF, heart failure; LODS, Logistic Organ Dysfunction System; LOS, Length of Stay; MI, Myocardial Infarction; MV, Mechanical Ventilation; NOAC, New-oral-anticoagulants; OASIS, Oxford Acute Severity of Illness Score; PLT, platelet count; RBC, red blood cell count; SAPS II, Simplified Acute Physiology Score

II; Scr, Serum creatinine; SIRS, Systemic Inflammatory Response Syndrome; SMD, Standardized Mean Difference; SOFA, Sequential Organ Failure Assessment; UV, Urine volume; WBC, white blood cell count.

Table S3. Multi-model regression analysis of secondary outcomes.

	90-day mortality			1-year mortality			In-hospital mortality		
	**HR**	**95%CI**	***p***	**HR**	**95%CI**	***p***	**OR**	**95%CI**	***p***
**Model 1**									
Non-combination	1.00	Reference		1.00	Reference		0.51	0.39- 0.68	
Combination	0.63	0.51-0.77	<.001	0.66	0.55- 0.80	<.001	0.51	0.39- 0.6	<.001
**Model 2**									
Non-combination	1.00	Reference		1.00	Reference		1.00	Reference	
Combination	0.62	0.50-0.77	<.001	0.65	0.54- 0.79	<.001	0.43	0.32- 0.58	<.001
**Model 3**									
Non-combination	1.00	Reference		1.00	Reference		1.00	Reference	
Combination	0.66	0.53-0.83	<.001	0.69	0.56- 0.85	<.001	0.44	0.31- 0.62	<.001
**Model 4**									
Non-combination	1.00	Reference		1.00	Reference		1.00	Reference	
Combination	0.66	0.53-0.83	<.001	0.69	0.56- 0.85	<.001	0.45	0.31- 0.65	<.001

Model 1: Crude. Model 2: Adjust: age, race, gender, BMI, ICU IOS. Model 3: Adjust for variables in Model 2 plus Temperature, admission GCS, APS III, BG, Blood potassium, Blood sodium, BUN, HR, LODS, MBP, OASIS, PLT, RBC, RR, Scr, SIRS, Spo2, SOFA, WBC, UV. Model 4: Adjust for variables in Model 3 plus AF, Alcohol Consumption, Antiplatelet drugs history, CHD, CLD, CKD, CRRT, Dementia, Diabetes, HF, Hypertension, MI, MV, Smoking, Statins, Thrombolysis, Warfarin, NOAC, Vasoactive Drugs.HR: Hazard Ratio, OR: Odds Ratio, CI: Confidence Interval.

Table S4 Mediating effect analysis of Bleeding in MAKI combined with IS patients.

Outcomes	Paths	Observed Coefficient	Sobel test		Bootstrap test			
			Z value	*p*	Bootstrap Standard Error	*p*	LLCI	ULCI
28-day mortality	Indirect effect	-0.014	0.668	0.503	0.024	0.570	0.024	0.035
	Direct effect	0.033			0.102	0.749	-0.167	0.233
	Total effect	0.019			0.100	0.849	0.177	0.215
90-day mortality	Indirect effect	-0.016	0.753	0.451	0.024	0.496	-0.066	0.031
	Direct effect	-0.012			0.105	0.908	-0.218	0.194
	Total effect	-0.029			0.103	0.783	-0.231	0.174
1-year mortality	Indirect effect	-0.026	0.997	0.318	0.025	0.305	-0.077	0.024
	Direct effect	-0.093			0.111	0.405	-0.311	0.125
	Total effect	-0.119			0.11	0.282	-0.334	0.096
In-hospital mortality	Indirect effect	0.006	0.367	0.713	0.028	0.840	-0.029	0.086
	Direct effect	-0.058			0.083	0.485	-0.22	0.104
	Total effect	-0.052			0.081	0.519	-0.211	0.106

LLCI, lower level for confidence interval; ULCI, upper level for confidence interval.

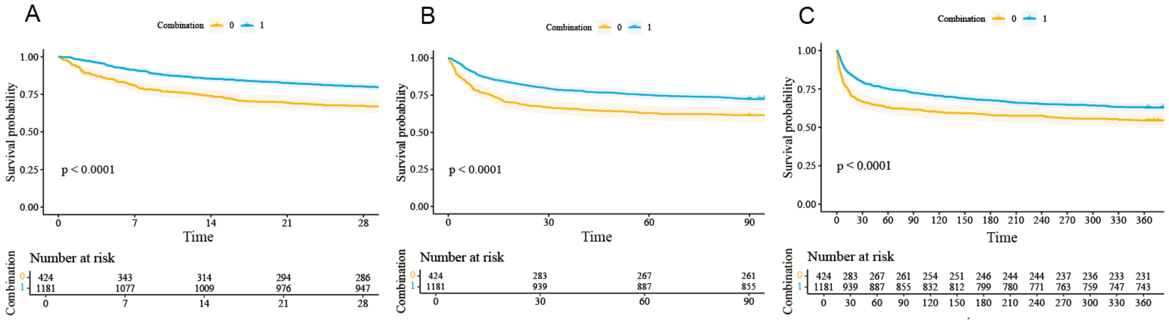

Figure S1. Kaplan Meier survival curves of combination group and non-combination group in patients with atrial fibrillation. (A) 28-day mortality. (B) 90-day mortality. (C) 1-year mortality.

Variables	HR(95%CI)	P	P for interaction
All patients	0.580 (0.450, 0.740)	<0.001	
Age			0.502
<65	0.470 (0.290, 0.790)	0.004	
≥65	0.580 (0.430, 0.780)	<0.001	
Gender			0.485
Female	0.620 (0.440, 0.880)	0.007	
Male	0.520 (0.360, 0.760)	<0.001	
Hypertension			0.041
No	0.880 (0.540, 1.430)	0.609	
Yes	0.490 (0.360, 0.660)	<0.001	
Diabetes			0.455
No	0.540 (0.390, 0.740)	<0.001	
Yes	0.660 (0.430, 1.010)	0.058	
Anticoagulants			0.021
No	0.490 (0.350, 0.670)	<0.001	
Yes	0.940 (0.600, 1.480)	0.788	
Atherosclerosis			0.031
No	0.520 (0.400, 0.690)	<0.001	
Yes	1.220 (0.590, 2.510)	0.589	
Aki			0.038
1	0.560 (0.430, 0.730)	<0.001	
2	0.870 (0.280, 2.660)	0.804	
3	4.240 (0.300, 45.320)	0.244	
AF			0.008
No	0.390 (0.270, 0.560)	<0.001	
Yes	0.790 (0.550, 1.130)	0.200	
Hemorrhage			0.004
No	0.680 (0.510, 0.890)	0.005	
Yes	0.230 (0.120, 0.450)	<0.001	

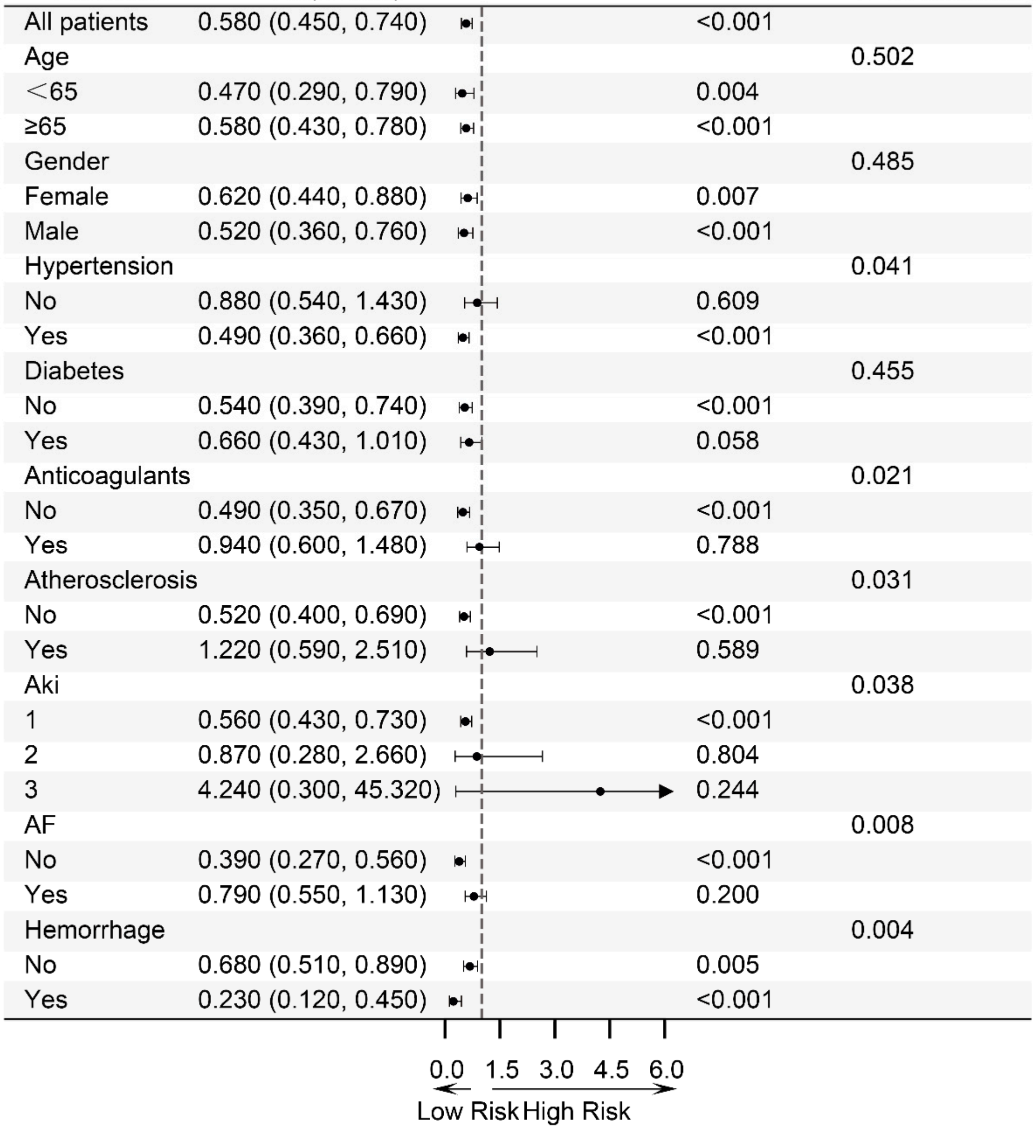

Figure S2. **Subgroup forest map of 90-day mortality.**

Variables	HR(95%CI)	P	P for interaction
All patients	0.620 (0.480, 0.790)	<0.001	
Age			0.104
<65	0.410 (0.250, 0.670)	0.004	
≥65	0.660 (0.490, 0.880)	0.005	
Gender			0.553
Female	0.650 (0.470, 0.910)	0.013	
Male	0.560 (0.390, 0.810)	0.002	
Hypertension			0.122
No	0.880 (0.540, 1.430)	0.609	
Yes	0.490 (0.360, 0.660)	<0.001	
Diabetes			0.190
No	0.540 (0.400, 0.740)	<0.001	
Yes	0.770 (0.510, 1.160)	0.209	
Anticoagulants			0.009
No	0.500 (0.360, 0.680)	<0.001	
Yes	0.990 (0.660, 1.490)	0.964	
Atherosclerosis			0.084
No	0.570 (0.440, 0.740)	<0.001	
Yes	1.120 (0.550, 2.290)	0.758	
Aki			0.040
1	0.610 (0.480, 0.790)	<0.001	
2	0.620 (0.220, 1.740)	0.361	
3	4.240 (0.300, 45.320)	0.244	
AF			0.008
No	0.420 (0.300, 0.600)	<0.001	
Yes	0.830 (0.580, 1.190)	0.318	
Hemorrhage			0.104
No	0.670 (0.510, 0.870)	0.003	
Yes	0.380 (0.200, 0.710)	0.003	

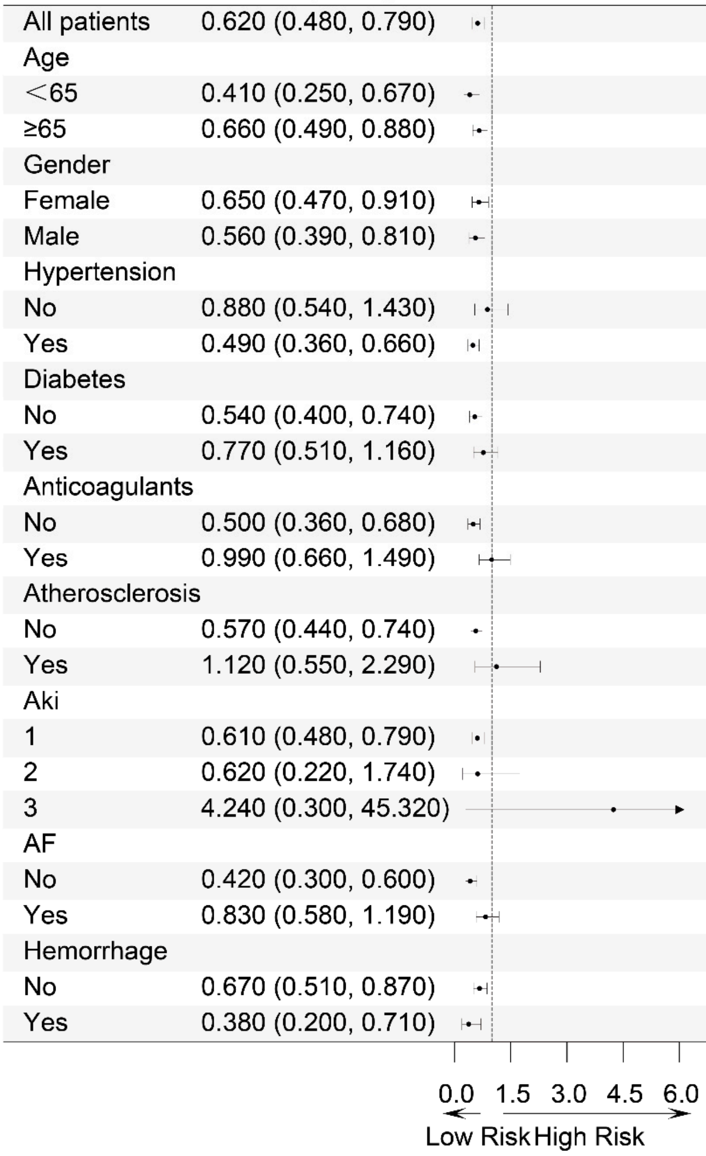

Figure S3. **Subgroup forest map of 1-year mortality.**

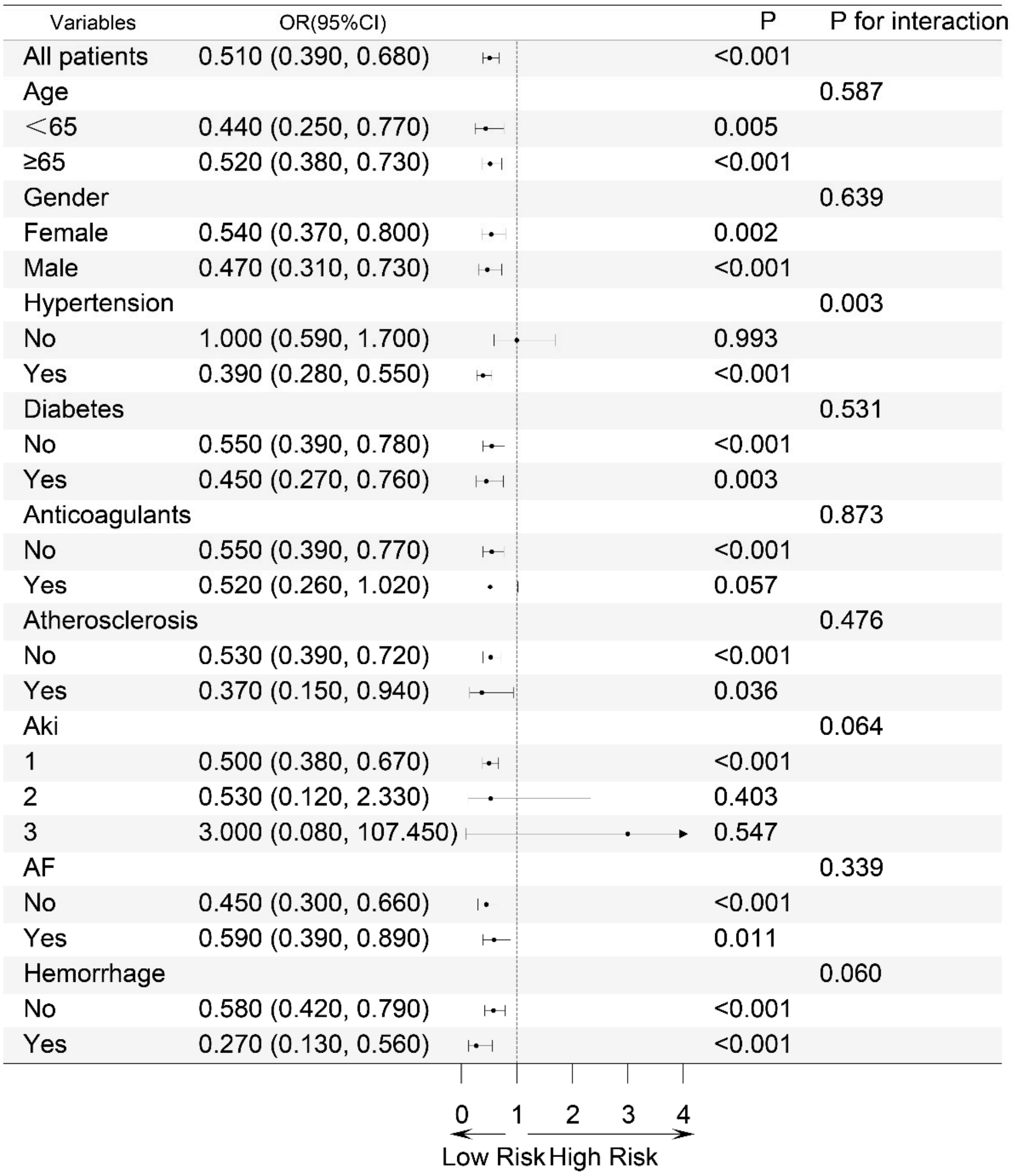

Variables	OR(95%CI)	P	P for interaction
All patients	0.510 (0.390, 0.680)	<0.001	
Age			0.587
<65	0.440 (0.250, 0.770)	0.005	
≥65	0.520 (0.380, 0.730)	<0.001	
Gender			0.639
Female	0.540 (0.370, 0.800)	0.002	
Male	0.470 (0.310, 0.730)	<0.001	
Hypertension			0.003
No	1.000 (0.590, 1.700)	0.993	
Yes	0.390 (0.280, 0.550)	<0.001	
Diabetes			0.531
No	0.550 (0.390, 0.780)	<0.001	
Yes	0.450 (0.270, 0.760)	0.003	
Anticoagulants			0.873
No	0.550 (0.390, 0.770)	<0.001	
Yes	0.520 (0.260, 1.020)	0.057	
Atherosclerosis			0.476
No	0.530 (0.390, 0.720)	<0.001	
Yes	0.370 (0.150, 0.940)	0.036	
Aki			0.064
1	0.500 (0.380, 0.670)	<0.001	
2	0.530 (0.120, 2.330)	0.403	
3	3.000 (0.080, 107.450)	0.547	
AF			0.339
No	0.450 (0.300, 0.660)	<0.001	
Yes	0.590 (0.390, 0.890)	0.011	
Hemorrhage			0.060
No	0.580 (0.420, 0.790)	<0.001	
Yes	0.270 (0.130, 0.560)	<0.001	

Figure S4. **Subgroup forest map of in-hospital mortality.**

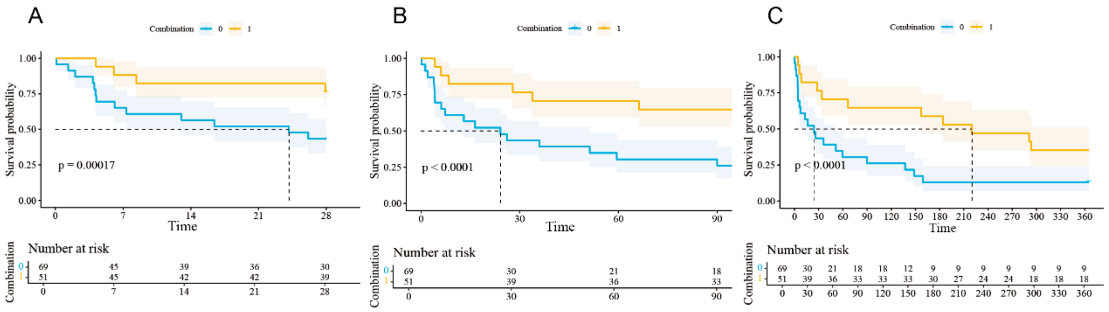

Figure S5. Kaplan Meier survival curves of combination group and non-combination group in patients without acute kidney injury. (A) 28-day mortality (B) 90-day mortality (C) 1-year mortality.
